# Prevalences and Interrelationships of Post COVID-19 Fatigue, Sleep Disturbances, and Depression in Healthy Young and Middle-Aged Adults

**DOI:** 10.3390/jcm13102801

**Published:** 2024-05-09

**Authors:** Changhwan Kim, Jae Young Moon, Sung Hyun Kim, Sun-Hyung Kim, Youjin Chang, Woo Hyun Cho, Won-Young Kim, Sun Jung Kwon, Ho Cheol Kim, Kwang Ha Yoo, Young Seok Lee

**Affiliations:** 1 Department of Internal Medicine, Hallym University Dongtan Sacred Heart Hospital, Hwaseong-si 18450, Republic of Korea; masque70@naver.com; 2 Division of Pulmonology and Critical Care Medicine, Department of Internal Medicine, Chungnam National University College of Medicine, Chungnam National University Sejong Hospital, Sejong 30099, Republic of Korea; diffable@hanmail.net; 3 Division of Pulmonary, Allergy, and Critical Care, Department of Internal Medicine, Inje University Busan Paik Hospital, College of Medicine, Busan 47392, Republic of Korea; handemoa@naver.com; 4 Division of Pulmonary and Critical Care Medicine, Department of Internal Medicine, Chungbuk National University Hospital, Chungbuk National University College of Medicine, Cheongju 28644, Republic of Korea; iinakaii@naver.com; 5 Division of Pulmonary and Critical Care Medicine, Department of Internal Medicine, Sanggye Paik Hospital, Inje University College of Medicine, Seoul 01757, Republic of Korea; yjchang0110@gmail.com; 6 Division of Allergy, Pulmonary and Critical Care Medicine, Transplant Research Center, Research Institute for Convergence of Biomedical Science and Technology, Pusan National University Yangsan Hospital, Yangsan 50612, Republic of Korea; popeyes0212@hanmail.net; 7 Department of Internal Medicine, School of Medicine, Pusan National University, Yangsan 46241, Republic of Korea; 8 Division of Pulmonary and Critical Care Medicine, Department of Internal Medicine, Chung-Ang University Hospital, Chung-Ang University College of Medicine, Seoul 06974, Republic of Korea; wykim81@cau.ac.kr; 9 Division of Respiratory and Critical Care Medicine, Department of Internal Medicine, Konyang University College of Medicine, Daejeon 35365, Republic of Korea; sjoongkwon@hanmail.net; 10 Department of Internal Medicine, Gyeongsang National University Changwon Hospital, Gyeongsang National University School of Medicine, Changwon 51472, Republic of Korea; hochkim@gnu.ac.kr; 11 Division of Pulmonary and Critical Care Medicine, Department of Internal Medicine, Konkuk University School of Medicine, Seoul 05030, Republic of Korea; 20010025@kuh.ac.kr; 12 Division of Pulmonary, Allergy, and Critical Care Medicine, Department of Internal Medicine, Korea University Guro Hospital, Seoul 08308, Republic of Korea

**Keywords:** post-COVID-19 condition, young age, socioeconomic burden, questionnaire, depression

## Abstract

**Background:** An evaluation of the persistence of symptoms following COVID-19 in economically active young and middle-aged adults is crucial due to its significant socioeconomic impact resulting from compromised work performance. **Methods:** A prospective, multicenter study at 12 South Korean hospitals from January to December 2022 involved telephone interviews along with validated questionnaires. **Results:** Among 696 participants with a median age of 32 and no prior diagnoses, 30% of participants experienced persistent fatigue, while 21.4% suffered from sleep disturbance at 6 months following infection. Additionally, approximately 25% of the participants exhibited depression that endured for up to 6 months. Symptomatic individuals at 3 months exhibited a significantly higher prevalence of persistent fatigue, sleep disturbances, and depression at 6 months compared to those who remained asymptomatic. Notably, sleep disturbance and persistent fatigue at 3 months emerged as significant independent predictors of the presence of depression at 6 months. **Conclusions:** Even among young and middle-aged healthy adults, prolonged fatigue, sleep disturbance, and depression exhibit a significant prevalence and persisted for up to 6 months. Therefore, implementing a workplace management protocol for these symptoms is essential to mitigate the socioeconomic burden caused by the impairment of work efficiency.

## 1. Introduction

Post-coronavirus disease 2019 (COVID-19) conditions, characterized by persistent symptoms exceeding 12 weeks after acute illness, poses a significant global health challenge due to its detrimental impact on daily functioning and quality of life [1,2]. Post-COVID-19 conditions is distinguished by its prolonged duration and diverse array of symptoms compared to other respiratory illnesses, such as influenza infection, despite exhibiting a similar prevalence of symptoms compared to other respiratory virus infections [3,4,5,6]. Fatigue and neuropsychiatric manifestations, such as sleep disturbances, anxiety, and depression, are the most frequently reported symptoms [7,8,9,10]; these symptoms significantly impair patient quality of life and ability to return to work [11,12,13,14,15,16]. Working individuals are particularly susceptible to long-term effects [17,18,19]. Less than half of previously full-time employees return to full-time work following COVID-19 infection, whereas approximately 18% quit their jobs altogether [20]. The compromised work performance in the working-age population due to long-term sequelae of COVID-19 translates into a significant socioeconomic burden, emphasizing the need for further studies on this condition in young and middle-aged healthy adults.

Previous studies on the post-COVID-19 conditions have predominantly relied on rudimentary inquiries, as exemplified by questions such as, “Has the participant experienced any symptoms since the acute phase of COVID-19 illness?” Such simplistic interrogations elicit primarily subjective sentiments that compromise diagnostic precision, hindering a precise understanding of the phenomenon. The use of meticulously validated questionnaires is crucial because they facilitate definitive diagnoses, the formulation of treatment strategies, and comparative analyses of changes in symptoms over defined intervals [21].

This study investigated the prevalences of fatigue, sleep disturbance, and depression in young and middle-aged healthy adults at 3 and 6 months after COVID-19 infection using validated questionnaires and explored interrelationships among these symptoms.

## 2. Materials and Methods

### 2.1. Study Design

A multicenter, prospective, observational study was conducted at 12 university-affiliated hospitals in South Korea between January 2022 and December 2022. The Omicron variant has become the dominant strain in South Korea since its initial detection on 25 November 2021 [22]. Recruitment notices were posted between January and June 2022. Participants who met the inclusion criteria and provided informed consent were included in the study. Enrolled individuals participated in telephone interviews conducted by a well-trained research nurse using validated questionnaires related to fatigue, sleep disturbance, and depression at 3 and 6 months after COVID-19 diagnosis. Fatigue, sleep disturbance, and depression were assessed using the following survey tools: the Korean version of the Schedule of Fatigue and Anergy/General Physician (SOFA/GP) that screens for prolonged and disabling fatigue [23]; the Korean version of the Pittsburgh Sleep Quality Index (PSQI) to evaluate sleep disturbance [24]; and the Korean version of the Patient Health Questionnaire-9 (PHQ-9) to assess depression [25,26]. To ensure the accuracy of all interviews, an online survey incorporating the questionnaires (e.g., SOFA/GP, PSQI, and PHQ-9) was administered to all patients, and the results were cross-verified in later telephone interviews. When the response to the online survey and the telephone interview differed, the research nurse asked the patient why the discrepancy had arisen and added the more accurate report to the database.

### 2.2. Ethics Statement

The study was approved by the institutional review boards of all participating hospitals (Table S1) and was conducted in accordance with the Declaration of Helsinki. Written informed consent was obtained from all participants after the researcher explained the study details directly before enrollment. Telephone surveys were administered according to a predetermined schedule thereafter.

### 2.3. Subjects

The inclusion criteria for participants were as follows: age ≥ 18 years and <60 years; a diagnosis of COVID-19 using a real-time polymerase chain reaction test during the study period; and a complete response to a telephone interview at 3 and 6 months post-COVID-19 infection. The exclusion criteria were failure to obtain informed consent; the presence of a comorbidity, such as asplenia, cancer, chronic heart disease, hypertension, chronic kidney disease, chronic liver disease, chronic lung disease, chronic neurological disorder, diabetes, immunodeficiency, tuberculosis, or psychiatric disease; or missing a telephone interview at 3 or 6 months post-COVID-19 infection.

### 2.4. Outcome Variables

The primary outcome was the prevalence of fatigue, sleep disturbances, and depression subsequent to COVID-19 infection in healthy individuals. The secondary outcome was the interrelationship between these symptoms following COVID-19 infection.

### 2.5. Questionnaires Used in this Study

The Korean version of the SOFA/GP is a well-validated tool for screening prolonged and disabling fatigue in community and primary care settings. This questionnaire includes 10 items; a cumulative score ≥ 3 indicates a diagnosis of prolonged, disabling fatigue. The Cronbach’s alpha coefficient for the SOFA/GP was 0.82 [23]. The Korean version of the 18-question PSQI is a well-validated index of sleep quality, sleep onset latency, sleep duration, sleep efficiency, sleep disturbance, use of sleeping medication, and daytime dysfunction, and a total score greater than 8.5 indicates poor sleep quality. The Cronbach’s alpha coefficient for PSQI was 0.84 [24]. The Korean version of the PHQ-9 is a well-validated screening tool consisting of nine questions. Out of a maximum of 27 points, a score of 1–4 points indicates a normal mental state, 5–9 indicates mild depression, 10–19 indicates moderate depression, and 20–27 indicates severe depression. Patients with mild depression require follow-up, and patients with moderate to severe depression require treatment. The Cronbach’s alpha coefficient for the PHQ-9 was 0.81 [25,26]. Note that permission to use each of these questionnaires was obtained from the copyrighted authors.

### 2.6. Statistical Analysis

Descriptive data are presented as median (25th to 75th percentile) or numbers and percentages. Proportional contributions of each questionnaire (e.g., SOFA/GP, PSQI, PHQ-9) at 3 and 6 months were compared using the McNemar test. The mean score of items in the PSQI at 3 months and at 6 months were compared using a paired *t*-test. Fisher’s exact test or a linear association test was used to determine the proportion of post-COVID-19 fatigue, sleep disturbances, and depression at 6 months, categorized by the number of symptoms at 3 months. In addition, logistic regression analysis was used to identify risk factors that independently predict depression at 6 months after COVID-19 infection. Independent variables and those with *p*-values < 0.1 in univariate analyses were included in multivariate analyses. The data are presented as adjusted odds ratios (ORs) with 95% confidence intervals (CIs). All statistical analyses were performed using SPSS software (ver. 20.0, IBM Corp., Armonk, NY, USA)

## 3. Results

### 3.1. Clinical Characteristics

**Figure 1:**
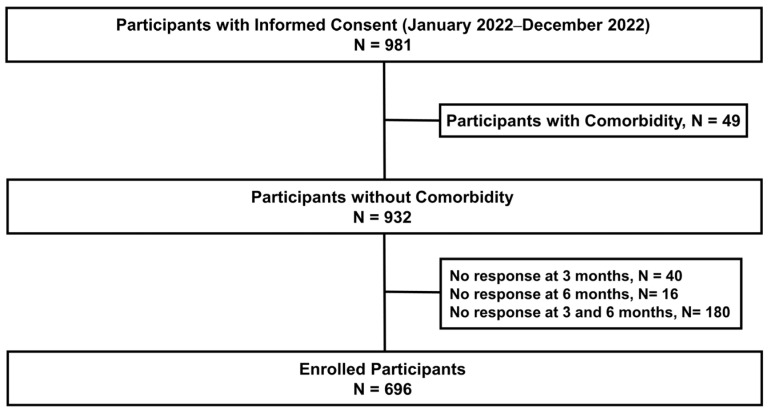
Flow chart of this study.

During the study period, 981 participants provided informed consent. Of these, 285 were excluded (49 participants with a comorbidity and 236 who declined a telephone interview at any or both timepoints (3 and 6 months)). Ultimately, 696 participants were included in this study (Figure 1).

The median age of the participants was 32 years, and 21% were male. The study group included 469 non-smokers (67.4%); 178 did not respond to the smoking questionnaire. The median duration of isolation at home during COVID-19 infection was 6 days. In total, 33 participants (4.7%) were reinfected with COVID-19 and 97.6% had received the COVID-19 vaccine. None of the participants were diagnosed with any disease prior to enrollment (Table 1).

#### 3.1.1. The Comparison of the Prevalence of Prolonged Fatigue among Participants at 3 and 6 Months Post-COVID-19 Infection

**Figure 2:**
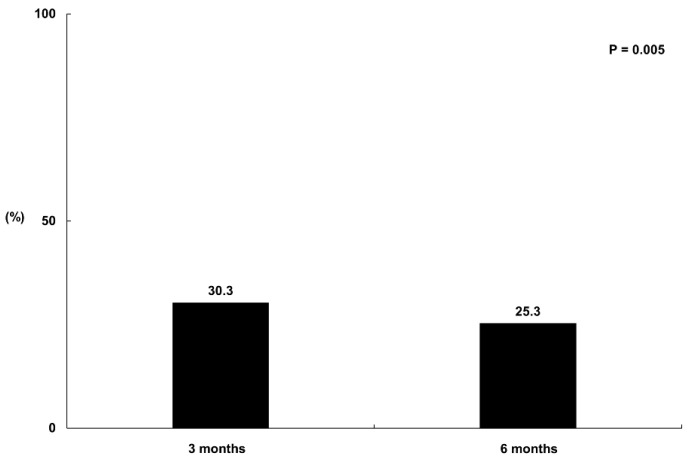
The proportion of participants with prolonged and disabling fatigue at 3 and 6 months after COVID-19 infection. The proportional contributions of SOFA/GP at 3 and 6 months were compared using the McNemar test.

The prolonged and disabling fatigue of the participants was evaluated using the Korean version of the SOFA/GP questionnaire. According to the responses, 211 participants (30.3%) and 176 (25.3%) suffered from prolonged and disabling fatigue at 3 and 6 months, respectively; the proportion suffering from prolonged and disabling fatigue decreased significantly at 6 months (*p* = 0.005; Figure 2).

Of the 10 items on the questionnaire, the proportion reporting “poor concentration”, “sleep for a long time”, and “muscle aches after activity” decreased by a statistically significant amount at 6 months compared to 3 months. However, approximately 30% of participants still complained of “being tired for a long time after activity”, “muscle tiredness after activity”, and “sleeping for a long time” at 6 months after COVID-19 infection (Figure S1).

#### 3.1.2. The Comparison of the Prevalence of Sleep Disturbance among Participants at 3 and 6 Months Post-COVID-19 Infection

**Figure 3:**
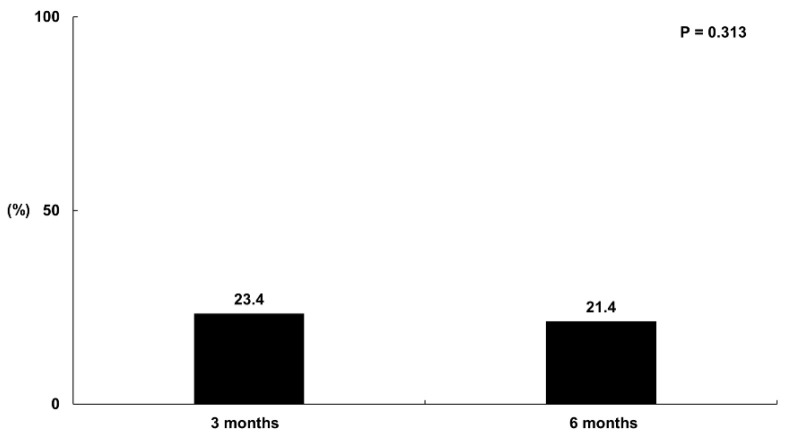
The proportion of participants with sleep disturbance at 3 and 6 months after COVID-19 infection. The proportional contributions of PSQI at 3 and 6 months were compared using the McNemar test.

Sleep disturbances were evaluated using the Korean version of the PSQI questionnaire. In all, 163 participants (23.4%) at 3 months reported suffering from sleep problems. At 6 months, this had decreased but not significantly so (149 participants, 21.4%); *p* = 0.313; Figure 3).

Among the seven items, daytime dysfunction had significantly improved at 6 months compared to 3 months; additionally, sleep latency and sleep disturbance also improved (Table S2).

#### 3.1.3. The Comparison of Depression Prevalence and Severity among Participants at 3 Months and 6 Months Post-COVID-19 Infection

**Figure 4:**
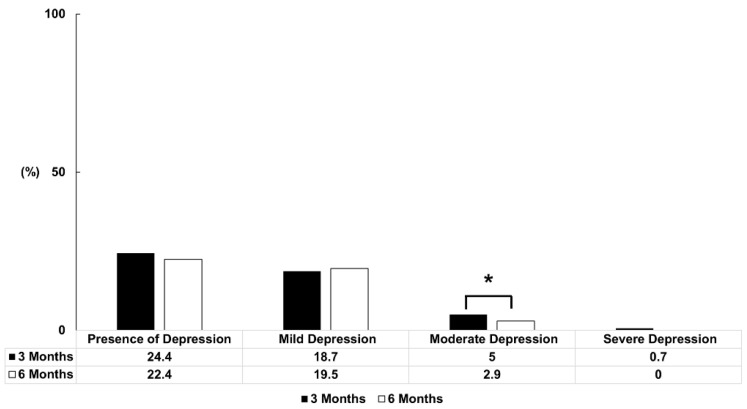
The proportion of participants with depression at 3 and 6 months after COVID-19 infection. The proportional contributions of PHQ-9 at 3 and 6 months were compared using the McNemar test. At 6 months, the change in the proportion of individuals with depression was not statistically significant. However, there was a statistically significant decrease in the number of individuals with moderate to severe depression requiring treatment compared to 3 months. * Statistically significant.

The presence and severity of depression were evaluated using the Korean version of the PHQ-9 questionnaire. Responses showed that 170 participants (24.4%) suffered from depression at 3 months, with the number falling to 156 (22.4%) at 6 months; the change was not statistically significant (*p* = 0.203; Figure 4).

Overall, 18.7% reported experiencing mild depression at 3 months, 5% had moderate depression, and 0.7% had severe depression. At 6 months, the values were 19.5%, 2.9%, and 0%, respectively. The change was statistically significant for moderate depression (*p* = 0.016; Figure 4). With regard to the need for treatment of depression, 5.7% of participants required interventions 3 months after COVID-19 infection; however, this proportion was significantly lower at 6 months (5.7% vs. 2.9%; *p* = 0.003).

#### 3.1.4. The Interrelationship among Post-COVID-19 Fatigue, Sleep Disturbances, and Depression at 3 and 6 Months Based on the Number of Symptoms

**Figure 5:**
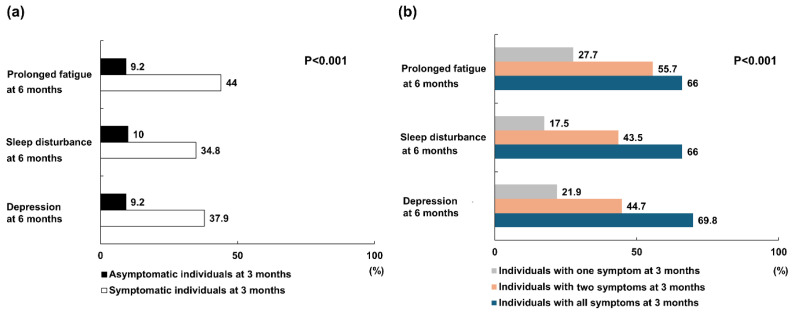
Prevalence of prolonged fatigue, sleep disturbances, and depression at 6 months based on presence and number of symptoms at 3 months. (**a**) The proportion of each symptom at 6 months between asymptomatic and symptomatic individuals at 3 months. Symptomatic individuals at 3 months exhibited a significantly higher prevalence of persistent fatigue, sleep disturbances, and depression at 6 months compared to those who remained asymptomatic. (**b**) The proportion of each symptom at 6 months according to the number of symptoms reported at 3 months. The prevalence of each symptom increased proportionally with the number of symptoms reported at the 3-month assessment.

At 3 months post-COVID-19 infection, 373 individuals (53.6%) were asymptomatic, whereas 155 (22.3%) reported one, 115 (16.5%) reported two, and 53 (7.6%) reported all three symptoms (e.g., prolonged fatigue, sleep disturbance, and depression). Of asymptomatic individuals at 3 months, 9.2%, 10% and 9.2% experienced prolonged fatigue, sleep disturbance and depression at 6 months, respectively. Conversely, 44%, 34.8% and 37.9% of symptomatic individuals at 3 months experienced prolonged fatigue, sleep disturbances and depression at 6 months, respectively (*p* < 0.001; Figure 5a). Among individuals with one symptom at 3 months, 27.7% experienced prolonged fatigue, 17.5% experienced sleep disturbance and 21.9% reported sleep disturbances at 6 months. Similarly, of those with two symptoms, 55.7%, 43.5% and 44.7% experienced prolonged fatigue, sleep disturbance and depression, respectively. Among individuals with all symptoms at 3 months, 66% experienced prolonged fatigue, 66% experienced sleep disturbance and 69.8% reported sleep disturbances at 6 months (*p* < 0.001; Figure 5b). Symptomatic individuals at 3 months exhibited a significantly higher prevalence of persistent fatigue, sleep disturbances, and depression at 6 months compared to those who remained asymptomatic. Moreover, the prevalence of these symptoms increased proportionally with the number of symptoms reported at the 3-month assessment.

**Figure 6:**
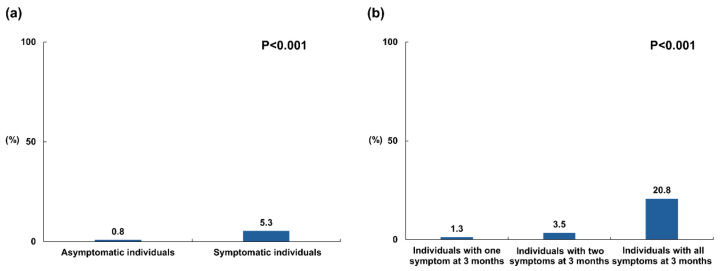
Prevalence of depression requiring intervention at 6 months based on the presence and number of symptoms at 3 months. (**a**) The proportion of depression requiring treatment at 6 months between asymptomatic and symptomatic individuals at 3 months. Symptomatic individuals at 3 months exhibited a significantly heightened prevalence of depression necessitating treatment at 6 months compared to those who remained asymptomatic. (**b**) The proportion of depression requiring treatment at 6 months according to the number of symptoms reported at 3 months. The prevalence of depression requiring treatment increased proportionally with the number of symptoms reported during the 3-month assessment.

With regard to the need for treatment of depression, 0.8% of asymptomatic individuals at 3 months and 5.3% of symptomatic individuals at 3 months required interventions 6 months after COVID-19 infection (*p* < 0.001; Figure 6a). At 6 months, treatment for depression was required by 1.3% of individuals with one symptom, 3.5% with two symptoms and, notably, 20.8% with all symptoms at 3 months (*p* < 0.001; Figure 6b).

#### 3.1.5. Risk Factors for Depression at 6 Months

Considering the significant impacts of depression on quality of life and mortality in young and middle-aged adults, we investigated the factors contributing to the persistence of depression at 6 months post-COVID-19 infection using logistic regression analysis. Univariate analyses indicated that female sex, the presence of prolonged fatigue at 3 months, and the presence of sleep problems at 3 months were significantly associated with depression at 6 months. Multivariate logistic regression (using backward elimination) revealed that the presence of prolonged fatigue at 3 months (OR, 3.54; 95% CI, 2.386–5.244; *p* < 0.001) and the presence of sleep disturbance (OR, 1.63; 95% CI, 1.071–2.486; *p* = 0.023) were significant risk factors for depression at 6 months (Table 2).

## 4. Discussion

This study assessed the prevalences of fatigue, sleep disturbance, and depression after COVID-19 in healthy young and middle-aged adults using well-validated questionnaires. Fatigue was assessed using the SOFA/GP questionnaire, which revealed that 25.3% of participants experienced persistent and disabling fatigue for up to 6 months, despite a decrease in prevalence compared to 3 months. In addition, 23.4% of participants at 3 months and 21.4% at 6 months suffered from sleep problems. Approximately 25% of participants had depression that lasted for up to 6 months. The prevalence of sleep disturbance and depression did not show a significant decrease at 6 months compared to their prevalence at 3 months. Approximately 40% of individuals with each of the aforementioned symptoms had persistent symptoms at 6 months, and the prevalence of these symptoms increased proportionally with the number of symptoms reported at the 3-month assessment. The presence of sleep problems and prolonged and disabling fatigue at 3 months were significant independent predictors of the presence of depression at 6 months.

Several studies on post-COVID-19 conditions have yielded diverse findings, influenced by time since infection, acute disease severity, geographic region, and sociodemographic characteristics, such as age and sex [7,8,9,10]. Mukherjee et al. conducted a meta-analysis that revealed a higher prevalence of post-COVID-19 conditions in hospitalized patients (0.54) compared to non-hospitalized patients (0.34), with females exhibiting a slightly higher prevalence (0.49) than males (0.37). Additionally, the pooled prevalence of post-COVID-19 conditions was lower in the USA compared to Europe (0.31 vs. 0.44), whereas a higher pooled prevalence was observed in Asia (0.51) compared to other regions [7]. During the early stages of the COVID-19 pandemic, studies predominantly focused on disease severity among hospitalized patients due to their increased mortality rates. Studies in this period have demonstrated that patients requiring advanced oxygen support, such as ventilator or ECMO, exhibited a higher prevalence and more severe post-COVID-19 conditions compared to those on less-intensive oxygen support, such as nasal cannula or high-flow nasal cannula [27,28]. The emergence of the Omicron variant, characterized by high transmissibility and reduced severity, resulted in a significant proportion of COVID-19 cases presenting with no symptoms or mild illness among young and middle-aged adults. This improved survivorship has emphasized the concerning issue of post-COVID-19 conditions, a long-term complication that can negatively impact long-term health, quality of life, and mental well-being. Consequently, multiple studies have shifted their focus to non-hospitalized young patients with such symptoms [29,30,31,32].

Young and middle-aged adults with post-COVID-19 conditions represent a significant socioeconomic burden due to their impaired ability to return to work [16,17,18,19,33,34,35,36]. According to the U.S. Census Bureau, of the estimated 16 million working-age adults affected by this condition, 2 to 4 million had not yet returned to their workplaces, resulting in an annual economic loss of approximately $170 billion [20]. Even for those who return to work, persistent symptoms such as brain fog, fatigue, and sleep disorders can reduce work efficiency and concentration [16,17,18,19,33,34,35,36]. Our study demonstrates that these symptoms may further exacerbate the situation by contributing to anxiety and depression. Additionally, we evaluated the impact of post-COVID-19 conditions on young and middle-aged adults with no prior diagnosed medical conditions. We found that a significant proportion of healthy individuals experienced symptoms such as fatigue, sleep disturbance, and depression after COVID-19 infection. In addition, individuals with more than one symptom exhibited a four- to five-fold higher prevalence of symptoms at 6 months compared to asymptomatic individuals. Notably, 5.3% of individuals with more than one symptom experienced depression, indicating an antidepressant potential need for antidepressant treatment. Therefore, the implementation of a management protocol in the workplace is crucial for early detection of symptoms and improved well-being of working patients with post-COVID-19 conditions. This protocol should include measures such as offering flexible work hours, reducing workload, and providing access to support services aimed at alleviating symptoms and promoting the well-being of affected individuals.

The precise mechanisms underlying the association between COVID-19 and neuropsychiatric problems remain unclear. Several hypotheses have been proposed in this regard. One possibility is that the virus itself or its shed particles may directly infect brain cells, leading to symptoms. Furthermore, COVID-19 can induce the release of cytokines, such as interleukin-1β, interleukin-6, and tumor necrosis factor-α, which may lower serotonin levels by altering its biosynthesis, release, and reuptake. Finally, environmental factors, such as isolation, insomnia, and decreased work efficacy, following COVID-19 infection can contribute to neuropsychiatric problems [37,38,39,40]. Further studies are needed to elucidate the precise mechanisms and establish treatment strategies for reducing post-COVID-19 conditions.

The strength of our study lies in its utilization of validated questionnaires to evaluate post-COVID-19 conditions. Use of a validated questionnaire is important; simple questions do not allow accurate diagnosis. If a validated questionnaire is used, a more accurate diagnosis is possible compared to a diagnosis based on responses to simple questions. In addition, in the case of depression, we were able to establish a treatment plan according to the severity of depression as indicated by the results obtained using the PHQ-9 questionnaire.

This study had some limitations, including selection bias in the recruitment of subjects. Participants in this study were likely to be very concerned about their health or to have symptoms after COVID-19 infection. Therefore, the proportion of symptoms may have been overestimated compared to the general population. Second, the proportion of men among the participants was small, which makes it impossible to effectively analyze any differences between women and men. Third, the proportion of participants aged 50 to 59 years was relatively small compared to the other age groups. Finally, the lack of pre-infection data using the same questionnaire limited our ability to compare post-COVID-19 conditions with a pre-infection baseline. If the study population consists of individuals with the disease, it is technically feasible to gather pre-infection data using the same questionnaire through follow-up procedures at our hospital. However, it was unfeasible considering that the primary focus of our study was post-COVID-19 conditions in a healthy young population. Despite these limitations, this study remains the first to evaluate post-COVID-19 conditions after infection in economically active populations free of pre-existing illness using validated questionnaires.

In conclusion, even among young and middle-aged healthy adults, prolonged fatigue, sleep disturbance, and depression exhibit a significant prevalence and persisted for up to 6 months. Notably, prolonged fatigue and sleep disturbances at 3 months were associated with depression at 6 months. These persistent symptoms can significantly impair work efficiency and hinder return to work, leading to a significant socioeconomic burden. Therefore, the implementation of a workplace management protocol is necessary to alleviate this burden. Considering our relatively small sample size of Korean patients, further multinational studies that use validated questionnaires in larger cohorts are needed to validate these findings.

## Figures and Tables

**Table 1:** Clinical characteristics of this study.

Variables	Participants (N = 696)
Age *	32 (27–40)
20–29	268 (38.5)
30–39	240 (34.5)
40–49	140 (20.1)
50–59	48 (6.9)
Male sex	146 (21)
Body mass index *	22 (20–25)
Smoking history	
Current smoker	18 (2.6)
Ex-smoker	31 (4.5)
Non-smoker	469 (67.4)
No response	178 (25.6)
Isolation period *	6 (5–6)
Re-infection	33 (4.7)
COVID-19 vaccination before COVID-19 infection	679 (97.6)
Booster COVID-19 vaccination	569 (81.8)

Abbreviation: COVID-19, coronavirus 2019. * Data are presented as median (25th to 75th percentile). Other variables are presented as number (%).

**Table 2:** Risk factors for the presence of depression at 6 months after COVID-19 infection using logistic regression analysis.

Variables	Odds Ratio	95% Confidence Interval	*p* Value
**Univariate analysis**			
Age	1.01	0.991–1.031	0.299
Female sex	2.07	1.242–3.445	0.005
Body mass index	0.98	0.927–1.036	0.482
Isolation period	1.08	0.939–1.233	0.294
Re-infection	1.77	0.840–3.740	0.133
Presence of prolonged fatigue at 3 months	4.23	2.911–6.158	<0.001
Presence of sleep disturbance at 3 months	2.46	1.666–3.627	<0.001
**Multivariate analysis**			
Presence of prolonged fatigue at 3 months	3.54	2.386–5.244	<0.001
Presence of sleep disturbance at 3 months	1.63	1.071–2.486	0.023

Multivariate logistic regression analysis using backward elimination was performed to investigate risk factors affecting depressive mood at 6 months after COVID-19 infection, after adjusting for 3 variables (female sex, presence of prolonged fatigue at 3 months, presence of sleep disturbance at 3 months) that are statistically significant in the univariate analysis.

## Data Availability

Data will be shared upon reasonable request to the corresponding author only after permission by the institutional review board of each participating hospital.

## Supplementary Materials

The following supporting information can be downloaded at: https://www.mdpi.com/article/10.3390/jcm13102801/s1, Table S1. IRB numbers of participating hospitals. Table S2. Comparison of average scores on the Korean version of the Pittsburgh Sleep Quality Index items between 3 months and 6 months. Figure S1. Variation of each item in the Schedule of Fatigue and Anergy/General Physician questionnaire at 3 months and at 6 months after COVID-19 infection.
