# Supplementary material for: Prevalences and Interrelationships of Post COVID-19 Fatigue, Sleep Disturbances, and Depression in Healthy Young and Middle-Aged Adults
Source: J Clin Med. 2024 May 9;13(10):2801. doi: 10.3390/jcm13102801 (PMC11122371; doi:10.3390/jcm13102801)
Supplement: Supplementary file 1 [file jcm-13-02801-s001.zip › jcm-2931982-supplementary.pdf]

Table S1. IRB numbers of participating hospitals

No.	Participating Hospital	IRB number	Approval date
1	Korea University Guro Hospital	2022GR0028	January 13, 2022
2	Jeju National University Hospital	2021-12-012	January 18, 2022
3	Chungnam National University Sejong Hospital	2022-01-017-001	February 4, 2022
4	Inje University Busan Paik Hospital	2022-01-008	January 24, 2022
5	Chungbuk National University Hospital	2021-12-020-002	January 27, 2022
6	Inje University Sanggye Paik Hospital	2021-12-008	January 21, 2022
7	Pusan National University Yangsan Hospital	05-2022-018	January 19, 2022
8	Chung-Ang University Hospital	2112-032-489	January 31, 2022
9	Korea University Anam Hospital	2022AN0028	January 17, 2022
10	Konyang University Hospital	2022-01-013	March 2, 2022
11	Gyeongsan National University Changwon Hospital	2022-01-017	February 3, 2022
12	Konkuk University	2022-01-006	February 24, 2022

Table S2. Comparison of average scores on the Korean version of the Pittsburgh Sleep Quality Index items between 3 months and 6 months.

Items	3 months	6 months	P value
Sleep quality	1.25±0.64	1.25±0.67	0.721
Sleep latency	1.48±0.86	1.42±0.86	0.086
Sleep duration	1.21±0.92	1.22±0.89	0.968
Habitual sleep efficiency	0.46±0.87	0.43±0.80	0.520
Sleep disturbance	1.02±0.43	0.99±0.51	0.087
Use of sleeping medication	0.08±0.41	0.08±0.39	0.744
Daytime dysfunction	0.99±0.88	0.82±0.83	<0.001

Data are presented as mean ± standard deviation. The mean scores of items in the Korean version of the Pittsburgh Sleep Quality Index at 3 months and at 6 months were compared using a paired t-test.

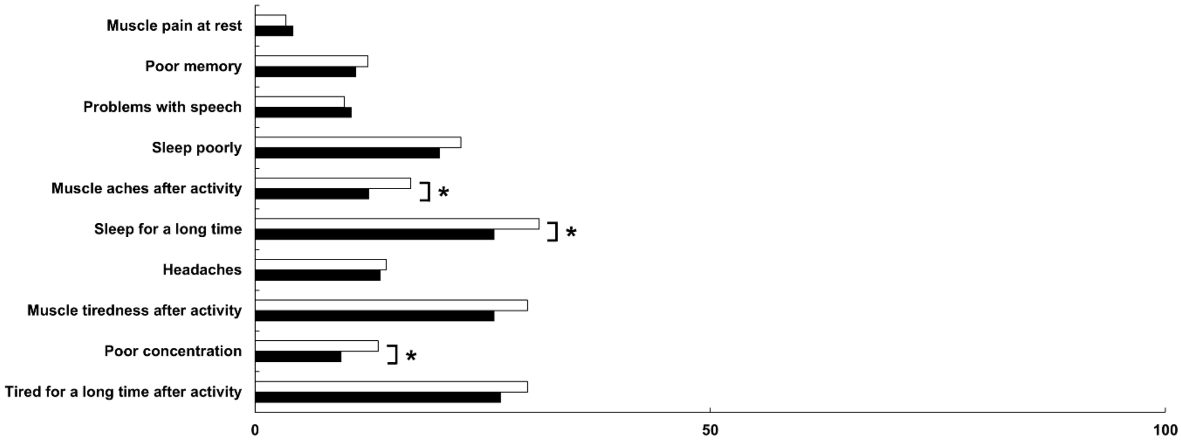

	Tired for a long time after activity	Poor concentration	Muscle tiredness after activity	Headaches	Sleep for a long time	Muscle aches after activity	Sleep poorly	Problems with speech	Poor memory	Muscle pain at rest
□3 months	29.9	13.5	29.9	14.4	31.2	17.1	22.6	9.8	12.4	3.4
■6 months	27	9.5	26.3	13.8	26.3	12.5	20.3	10.6	11.1	4.2

□3 months ■6 months

Figure S1. Variation of each item in the Schedule of Fatigue and Anergy/General Physician questionnaire at 3 months and at 6 months after COVID-19 infection.
